# Book and Pamphlet Notices

**Published:** 1858-04

**Authors:** 


### BOOK AND PAMPHLET NOTICES.

Human Histology, in its Relations to Descriptive Anatomy, Physiology and Pathology, with Four Hundred and Thirty-four Illustrations on Wood. By E. R. Peaslee, A.M., M.D., etc. etc. "Maxime in Minimis." Philadelphia: Blanchard & Lea. 1857.

The invention of the microscope opened to naturalists new fields of study, revealing forms both animate and inanimate, which had hitherto been entirely unknown; but it is chiefly in the world of life that this instrument has presented the most interesting as well as most useful results. It has become as necessary to the student of anatomy as his dissecting case; while, to the pathologist, it is almost a *sine qua non* in the investigation of the morbid elements, forces, forms and functions with which he has to do.

It has created, indeed, a new department of our science, occupying the intermediate ground between the coarser normal and morbid anatomy on the one hand, and rational physiology and pathology on the other. It is to this department that Dr. Peaslee has mainly devoted his work; while, at the same time, he has not ignored the intimate relation existing on the one hand between forms and elements, and on the other, between forms and functions. The work, indeed, comprises histology, combined with much that is valuable in organic chemistry, physiology and pathology.

The following plan of the work will enable our readers to form some opinion of what it comprises:

First, we have stœchology, comprising the classification and description of the simple chemical elements and of the immediate principles which enter into the composition of the fluids and tissues of the body.

The first division of this part is devoted to the simple chemical elements.

The second division to the immediate principles. These he divides into two groups; the first of which consists of those of mineral origin, and those formed within the body by dis-assimilation. The second group consists of organic or coagulable principles.

The second part of the work is devoted to histology; comprising, first, the simple forms of matter, membranes, fibres and cells; their development, distribution, functions and pathological conditions. Second, hygrology, or a description of the fluids of the body, including the lymph, chyle, and blood; the serous secretions and transudations; mucous and glandular secretions, and the cutaneous secretions. Third, the tissues, in the following order: Epithelium, nails, and hair; yellow fibrous tissue, white fibrous tissue, areolar tissue, adipose tissue, cartilage, osseous tissue and the bones, dental tissue, the teeth, muscular tissue and the muscles, nervous tissue and the structure of the nervous system, the membranes, the vascular system, the alimentary canal and its appendages, the urinary apparatus, the sexual organs, the respiratory organs, the blood, vascular glands, and the organs of the senses.

The illustrations are good, and add very much to the value of the work.

This volume meets the wants of the profession, and we heartily commend it to all those who would become thoroughly acquainted with the minute and beautiful structures of the human body. J——

A Manual of Medical Diagnosis, being an Analysis of the Signs and Symptoms of Disease. By A. W. Barclay, M.D., *Cantab. et Edin.*, Fellow of the Royal College of Physicians, Assistant Physician to St. George's Hospital, etc. etc. Philadelphia: Blanchard & Lea. For sale by W. B. Keen, Chicago.

Dr. Barclay's ample opportunities for practising and verifying physical diagnosis are well known to many of the profession. And he has made good use of his opportunities, as every one will attest who may avail themselves of the privilege of reading the above volume. Look at this volume, and then recur in your memory to the condition of this all-important branch of pathology twenty-five years ago, and say that the science of medicine does not keep pace with any of its sister sciences! With the appliances now available to penetrate the innermost recesses of man's nature — the ophthalmoscope, auroscope, stethoscope, urinometer, chemical tests, etc. etc., to say nothing of the more philosophical mode of handling the rational symptoms of disease—will show improvements in this department of pathology correspondingly great indeed. Dr. Barclay has brought all these improvements to bear upon the diseases included in his work with admirable aptitude and benefit. We can do nothing more with the space allowed us than to recommend our professional brethren to procure a copy, if they do not wish to fall behind in the forward rush of mind in this direction. It contains about 325 pages, much of which is in quite fine print, but it is executed in Blanchard & Lea's best style, so that it may be read with facility.

THE ANNIVERSARY DISCOURSE before the New York Academy of Medicine. By J. MARION SIMS, Surgeon to the Woman's Hospital.

On the 18th November, 1857, Dr. J. Marion Sims, Surgeon of the Woman's Hospital, New York, delivered an anniversary discourse before the New York Academy of Medicine on the subject of "Silver Sutures in Surgery," in which he claims, "THAT THE USE OF SILVER AS A SUTURE IS THE GREAT SURGICAL ACHIEVEMENT OF THE NINETEENTH CENTURY."

Dr. Sims claims to be the discoverer of the silver suture, and to have used it first, June 21st, 1849.

A claim so broad, brought forward by the alleged discoverer, in a century which has witnessed the discovery of the use of anesthetics in surgery, subcutaneous division of tendons, etc., is well calculated to create surprise among all those familiar with the history of surgical operations.

Dr. Sims did not invent the silver suture. We do not undertake to decide who invented it, but we know that Deiffenbach used it in his operations on the palate, but seems to have preferred sutures of leaden wire, which, since his time, have been very generally adopted. Dr. Metteucci, *American Jour. Med. Science*, vol. xxi., p. 216, in an excellent article on palate sutures, gives the preference to lead.

Dr. Sims, in this discourse, abandons the peculiarities of his operation as formerly published, in every essential respect. These peculiarities consisted in the use, 1st, of a "clamp" or metallic head fixed on the wire; 2d, in placing the patient on the face instead of on the back during the operation. At present, the only peculiarity claimed by Dr. Sims is in the use of silver wire instead of leaden wire. It is probable that further experience will induce him to follow the practice of Deiffenbach, and resort to the use of lead. Dr. Brainard has already used leaden wire in three cases of operation for vesico-vaginal fistula with success.

The use of the metallic head on the ligature, for the purpose of fetching the edges together, is not new, and had been tried long ago. In a case of cleft palate, operated by Sir Philip Crampton, Mr. Maclean applied them. They do not seem to have been of any particular service.

The object of this discourse seems to be mainly to reclaim priority in this operation against Dr. Bozeman, who had adopted and claimed to have improved on Dr. Sims' method. It appears to us that, in this reclamation, Dr. Sims has the show of justice on his side; but, perhaps, if both sides were heard, it might change the aspect of affairs.

While we applaud the enterprise and success which has originated and crowned the Woman's Hospital movement, we are not able to see the justice or wisdom of the claims set up by Dr. Sims. Perhaps he would conceive any suggestions as intrusions; but, at the risk of that, we take the liberty of stating, that the method recommended by Dr. Sims for introducing the stitches is exceedingly imperfect. There are several instruments by which this part of the operation can be much more easily done.

We would also suggest, that the use of a flat leaden wire will, in all probability, be found preferable.

Finally, it is due to the public and the profession, that such a report should be made of the cases treated in the Woman's Hospital, as to enable us to know to what extent the treatment therein used is superior to that in general use. For it is, we presume, the result of the experience of every surgeon who has undertaken the treatment of these cases, that moderate-sized openings, without loss of substance, are easily cured, and that destruction of the vesico-vaginal wall to a great extent is incurable. We should be pleased to learn where the line of demarkation runs in the cases treated in the Woman's Hospital.

In the March No. of the *North-American Medico-Chirurgical Review*, we find a paper by our friend, Dr. E. Read, Terre Haute, Ind., on the Mortality Statistics of the United States. "Mr. DeBow, the Superintendent of the Census Bureau, invoked the aid of Dr. Edward Jarvis, of Massachusetts, to classify the variously-named diseases which were found upon the marshal's returns." A large part of the diseases mentioned as the cause of deaths in the marshal's returns, were given in the popular instead of the scientific names, and as these vary in the diverse portions of our widely-extended country, a great deal of labor was necessary to translate them into significant and definite terms, and we agree with Dr. Read, that this might and ought to have been, but certainly was not done.

Dr. Jarvis was no doubt as capable as most men, whose residence has been during his whole life in an unchanging population; and probably the only way to arrive at completely correct results, in regard to the popular nomenclature of disease, would be to consult physicians resident in the different sections of our Union. We think, there are but few men to whom such a course would not have suggested itself. As specimens of the errors committed by Dr. Jarvis, we submit a few quotations made by Dr. Read.

"Diseases of the eye may involve diseases of the brain; but otherwise is not fatal. It should, therefore, be included in diseases of the brain and nervous system." Mumps comes under the same category as the last, (diseases of the eyes). It may be connected with disease of the brain, and it may co-exist with other and fatal diseases. It would be safer to put these in the unknown. Onanism, possibly, but not probably, was a cause of death. It wastes life, and produces other disorders. Cases under this head, should be put under the unknown. Fever, bilious, is a most vague and uncertain term. In some regions, it seems to be used for all sorts of fever, and the word bilious, for all sorts of difficulties of the digestive organs. Gastric fever is, probably, intended for inflammation of the stomach; yet it is, in many places, used as vaguely in respect to fevers, as the word bilious.

"Winter fever is more vague than either of the last two. It may mean bilious, or typhus, or synochus. These three, with specific names, convey no specific idea, and they should be included under the general name of fevers, without further description." It will only be necessary for the western physician to glance over the above instances of misapplication of terms to disease by Dr. Jarvis, to indicate to him the propriety of Dr. Read's criticism.

We advert to this paper in order to indorse all that Dr. Read has said upon the subject, and we regret that it cannot be included in the same document with the report, so that it might be used in correcting as historical data any erroneous impression that, as things now stand, may enter into our future history.

Since writing the above portion of this article, we have received the paper from its author in separate form. We are glad to see this indication of the Doctor's intention to circulate it still more extensively, as it certainly is desirable to set on foot some means of placing so important a correction on record.

### NEW JOURNALS.

We have received No. 1, Vol. I., of a new journal, published in Detroit, Mich., edited by Professors A. B. Palmer, Moses Gunn, and Mr. Frederick Stearns, entitled "THE PENINSULAR AND INDEPENDENT JOURNAL," which, as its name implies, is the result of an amalgamation of the two excellent journals published there. We hail this good-looking neighbor with our best wishes for a bright success and future prosperity. In looking it over, however, we notice quite an omission, in the copy on our table, beginning at page 32, and ending at page 49; leaving out the *Salutatory*, *Editorial Individuality*, and some other *less important* articles indicated in the table of contents. Their place is supplied by a repetition of matter, beginning with page 17. We are sorry to see the first-born of this happy editorial union thus deformed by the bungling operatives who officiated at its appearance in this world of light, and we earnestly hope that is no evidence of an inherited diathesis, destined to affect the whole future progeny. We trust the talented Professor of Surgery, who is noted for his ability in mending matters, will not delay a suitable operation—while his beloved offspring is yet young and impressible—to prevent it from growing into maturity with its parts in anywise deficient or out of joint; while the indefatigable Professor of Materia Medica and Diseases of Women and Children, will undoubtedly be careful to eradicate any parental taint that may possibly linger in the blood of its immediate progenitors.

Another instance of amalgamation has given origin to our excellent cotemporary, "THE CINCINNATI LANCET AND OBSERVER," edited by Professors Mendenhall and Murphy, of the Ohio Medical College, and Edward B. Stephens, M. D. We remember this trio from days long gone by, and with the warmth of fraternal interest, assure our brethren of the profession, that the *Lancet and Observer* will be found all right in time of need.

"THE PACIFIC MEDICAL AND SURGICAL JOURNAL" is among our exchanges. From its appearance, we should infer that its editor and publishers would be rewarded, if the profession of California can appreciate honest labor in the cause of our science. It is edited by Drs. J. B. Trask and D. Wooster, of San Francisco.

### EDITORIAL CHANGES.

Dr. J. Dickson Bruns has taken the entire control of the *Charleston Medical Journal.* This journal has been heretofore one of our best exchanges, and we confidently expect it to increase in merit and usefulness under its present management.

Drs. R. C. Foster and George S. Blackie have added their labors to the herculean strength of the senior editor of the *Nashville Journal of Medicine and Surgery.* With such a team, that journal cannot fail to "go." We welcome them to the great family of editors, and hope their "hearthstones may ever remain warm."

